# Influence of *Bacillus thuringiensis* and avermectins on gut physiology and microbiota in Colorado potato beetle: Impact of enterobacteria on susceptibility to insecticides

**DOI:** 10.1371/journal.pone.0248704

**Published:** 2021-03-24

**Authors:** Olga V. Polenogova, Yury A. Noskov, Olga N. Yaroslavtseva, Natalya A. Kryukova, Tatyana Alikina, Tatyana N. Klementeva, Jelizaveta Andrejeva, Viktor P. Khodyrev, Marsel R. Kabilov, Vadim Yu Kryukov, Viktor V. Glupov

**Affiliations:** 1 Institute of Systematics and Ecology of Animals, Siberian Branch of the Russian Academy of Sciences, Novosibirsk, Russia; 2 National Research Tomsk State University, Tomsk, Russia; 3 Institute of Chemical Biology and Fundamental Medicine, Siberian Branch, Russian Academy of Sciences, Novosibirsk, Russia; University of Tennessee, UNITED STATES

## Abstract

Gut physiology and the bacterial community play crucial roles in insect susceptibility to infections and insecticides. Interactions among Colorado potato beetle *Leptinotarsa decemlineata* (Say), its bacterial associates, pathogens and xenobiotics have been insufficiently studied. In this paper, we present our study of the survival, midgut histopathology, activity of digestive enzymes and bacterial communities of *L*. *decemlineata* larvae under the influence of *Bacillus thuringiensis* var. *tenebrionis* (*morrissoni*) (*Bt*), a natural complex of avermectins and a combination of both agents. Moreover, we estimated the impact of culturable enterobacteria on the susceptibility of the larvae to *Bt* and avermectins. An additive effect between *Bt* and avermectins was established regarding the mortality of the larvae. Both agents led to the destruction of midgut tissues, a decrease in the activity of alpha-amylases and alkaline proteinases, a decrease in the *Spiroplasma leptinotarsae* relative abundance and a strong elevation of Enterobacteriaceae abundance in the midgut. Moreover, an elevation of the enterobacterial CFU count was observed under the influence of *Bt* and avermectins, and the greatest enhancement was observed after combined treatment. Insects pretreated with antibiotics were less susceptible to *Bt* and avermectins, but reintroduction of the predominant enterobacteria *Enterobacter ludwigii*, *Citrobacter freundii* and *Serratia marcescens* increased susceptibility to both agents. We suggest that enterobacteria play an important role in the acceleration of *Bt* infection and avermectin toxicoses in *L*. *decemlineata* and that the additive effect between *Bt* and avermectin may be mediated by alterations in the bacterial community.

## Introduction

Colorado potato beetle *Leptinotarsa decemlineata* (Say) (Coleoptera: Crysomelidae) (CPB) is a cosmopolitan pest of *Solanaceae* with a high plasticity and high migratory ability [1]. Environmentally friendly bioinsecticides may be effectively included in the integrated management of the CPB population. Currently, bioinsecticides based on fungi, bacteria and their metabolites [2] and on plant extracts [3] and their semisynthetic derivatives are being actively developed to control the abundance of CPB. *Bacillus thuringiensis* (*Bt*) (Berliner, 1915) is the best-known base for most commercial bioinsecticides [4, 5]. The mechanism of *Bt* endotoxin (Cry) action is binding to specific receptors of the gut epithelial layer of invertebrates under alkaline conditions (Lepidoptera), followed by pore formation and cell lysis [6, 7]. However, Coleoptera have an acidic midgut, which likely suggests additional mechanisms for dissolution of the toxin [8–10]. Probably, in CPB cysteine and aspartic digestive proteases can participate in this process [11]. Their presence can act as a specificity factor and play a role in insect sensitivity to *Bt*. However, *Bt*-based insecticides have a low stability due to low resistance to various biotic and abiotic factors. This may lead to the need for repeated treatments or an increase in doses of bioinsecticides [12]. In addition to low stability, *Bt*-bioinsecticides have another significant drawback in the resistance of late-instar CPB larvae. Various natural metabolites may stabilize the activity of *Bt*-based insecticides. For example, several research groups have previously shown an additive effect between *Bt* and azadirachtin against different larval instars of *Helicoverpa armigera* (Lepidoptera) under laboratory conditions [13, 14].

Other prospective candidates for CPB control are avermectin-based products. The avermectin complex is represented by 16-membered macrocyclic polyketides (A1a, A2a, B1a, B2a, A1b, A2b, B1b and B2b) produced by the soil bacteria *Streptomyces avermitilis* and used as the basis of various commercial products [15, 16]. Under laboratory conditions, additive and synergistic effects between avermectins and entomopathogenic fungi in the mortality of CPB larvae have been shown [17–19]. Avermectins have a neurotoxic effect, and the mechanism of action on insects is the suppression of nerve and muscle conduction by binding to gamma-aminobutyric acid receptors on nerve cells [20]. Depending on the dose, this leads to paralysis, nutritional failure and death [21]. Studies with cockroaches (*Blattella germanica*), bees (*Apis mellifera*) and mosquitoes of the *Culex* and *Anopheles* genera have shown that the effect of sublethal doses of different avermectin compounds leads to the destruction of the Malpighian tubules and to the fusion of the gut epithelial layer accompanied by the destruction of digestive cells and tissues [22–24]. Median lethal doses of avermectins reduce the intensity of food consumption by the CPB with a subsequent delay in the development of larvae [19].

The composition and level of enzyme activity in the insect gut play a significant role in the detoxification of xenobiotics [25]. In particular, a change in the spectrum of proteolytic enzymes may determine insect resistance to insecticides [26–28]. It is known that activation of *Bt* toxin occurs in the alkaline environment of the insect gut under limited proteolysis [6, 29] and leads to a change in the feeding behavior and the structure of the gut bacterial community [30]. Insect microbiota contribute to the stable functioning of many physiological systems and mediate resistance to insecticides and pathogenic microorganisms [31–34]. The shifts in the microbial community may influence the susceptibility of insects to insecticides, including *Bt*-based products. In particular, native microbiota are able to act as a protective barrier [35, 36], interact with pathogens synergistically or additively [31, 37, 38], and change their status from commensal to pathogenic by penetration of the insects’ hemocoel [39, 40]. In addition, it was shown that the axenous insect *Lymantria dispar* was less susceptible to *Bt* than native insects, but their susceptibility was increased after recolonization with *Enterobacter* sp. [31, 37].

Synthetic and natural insecticides can also lead to shifts in the abundance of certain groups of bacteria in insects [41–43]. For example, fipronil increases the abundance of *Lactobacillus* and decreases the abundance of enterobacteria in the gut of *Plutella xylostella* [34]. Importantly, the predominant group of CPB gut bacteria is Enterobacteriaceae [44–46], which, as mentioned above, can influence the development of *Bt* pathogenesis and susceptibility to insecticides.

In the present work, we hypothesized that avermectins and *Bt* can enhance each other’s action on the basis of increased damage to CPB gut tissues, changes in enzyme activity and shifts in the bacterial load and bacterial community. The objectives of the study included (i) assessment of the mortality dynamics of Colorado potato beetle larvae, (ii) analysis of the pathomorphology of the gut tissues, (iii) assessment of the activity of digestive enzymes, and (iv) changes in bacterial load and microbiome structure under the influence of *Bt*, avermectins and their combination. In addition, we assessed the effect of the predominant Enterobacteriaceae on larval susceptibility to *Bt* and avermectins.

## Materials and methods

### Insects, bacteria and insecticides

The experiments were carried out on Colorado potato beetle larvae collected from private potato fields free from *Bt* and avermectin treatments (Novosibirsk region, Russian Federation; 53°44′3.534″N, 77°39′0.0576″E). These potato fields were not located in protected areas and we did not need special permission to collect beetles. Landowners did not prevent access to the fields. Endangered or protected species were not used in the work.

The insects were kept in a ventilated laboratory room for a 12:12 h dark/light period at a constant temperature of 25°C. The insects were fed fresh foliage from the potato *Solanum tuberosum*. Fourth-instar larvae (2–4 h postmolt in IV instar) were used for the experiments.

The entomopathogenic bacteria *Bacillus thuringiensis* var. *tenebrionis* (*Bt*) (H8ab *morrisoni*), 2495K2 from the collection of entomopathogenic microorganisms at the Institute of Systematics and Ecology of Animals of Siberian Branch of Russian Academy of Sciences (ISEA SB RAS), were used for the infection. The bacteria were grown on nutrient agar (Himedia, Mumbai, India) at 28°C. The spores and crystals were collected with a sterile spatula and washed twice with 150 mM NaCl (6000 × g, 10 min). The concentration of spores and crystals was determined using a hemocytometer. In the suspension, the concentration of spores and crystals used for the treatment was 3 × 10^7^/mL. The ratio of spores to crystals was 1: 1. An industrial product based on avermectins, “Aktarophit” 1.8% (“ENZIM Group”, Ukraine), was used as a water solution with a concentration of active substance (B1a and B2a) of 1.125 μg/L.

### Treatments and bioassay

The treatment was performed by dipping potato leaves (fresh weight of 3 ± 0.5 g) into 5 mL of the *Bt* suspension, avermectin solution or their combination for 30 s. The leaves in the control treatment were treated with the same volume of distilled water. We did not use any additional wetting agents for the treatments of potato leaves. The plants were dried for one hour at room temperature and placed in ventilated 300-mL plastic containers with 12 CPB in each container. These containers were kept under temperature and light conditions as described above. Forty-eight hours posttreatment, and further daily, all leaves were replaced with untreated potato leaves. The mortality of the insects was assayed daily for 5 days. At least 20 replicates (one replicate = 12 larvae) for each treatment were used in this bioassay.

### Electron microscopy of midguts

At 48 h posttreatment, the larvae were subjected to cryoanesthesia at -4°C and dissected, after which the midguts were isolated, placed in a solution of 2% glutaraldehyde in 0.1 M Na-cacodylate buffer (pH 7.2) and maintained at 4°C for 24 h. Then, the samples were washed with cacodylate buffer, postfixed in 1% (w/v) OsO_4_ in the cacodylate buffer and stored at 4°C for one month. The samples were dehydrated in an ethanol series, embedded in an Epon-Araldite 812 mixture and sectioned with a Reichert Ultracut S ultratome (Leica, Nussloch, Germany). Thin sections were stained with uranyl acetate and lead citrate and observed with a Hitachi-300 or a JEM-100CX electron microscope.

### Sample preparation for enzymatic activity

At 48 h posttreatment, insect midguts were isolated on ice, the contents were removed, and the tissues were collected in tubes (1 sample = 2 midguts) with a prechilled 100 μL 0.1 M phosphate buffer (PB) containing 150 mM NaCl (pH 7.2) and phenylthiourea (4 mM). After mechanical homogenization, the samples were centrifuged for 5 min at 10000 × g at 4°C. The supernatants were used for analysis of enzymatic activity.

### Total alkaline proteolytic activity and alpha-amylase activity

Total proteases were measured according to the methods of Elpidina *et al*. [47] and Gatehouse *et al*. [48] using 0.5% azocasein as substrate (AppliChem, Germany). Thirty μL of sample was mixed with 250 μL of substrate solution (250 μL 0.5 mM Tris-HCl buffer containing 150 mM NaCl, pH 8.0). After 40 min incubation at 25°C, the enzymatic reaction was stopped by the addition of 250 μL of 20% trichloroacetic acid (TCA) (Labochem Ltd., Germany). The mixture was kept on ice for 10 min and centrifuged at 10000 × g for 10 min. Enzyme activity was determined spectrophotometrically at a 366 nm wavelength.

The α-amylase activity was measured by methods previously published by Bernfeld [49] and Bandani *et al*. [50]. Thirty μL of sample were incubated for 10 min at 27°C with 30 μL 0.1 M Tris-HCl buffer (pH 8.7) containing 1% soluble starch as the substrate. The reaction was stopped by the addition of 60 μL buffer stop solution (30% acid disodium salts and 1.6% dinitrosalicylic acid solubilized in 1.6% hydroxide sodium) and heated in boiling water for 10 min. After cooling on ice for 5 min, absorbance was recorded at a 540 nm wavelength. Reactions without the enzyme extract were used as the control. Enzymes activity was measured in units of the transmission density (∆A) of the incubation mixture during the reaction per 1 min and 1mg of protein. The protein concentration was determined using the method described by Bradford [51]. Bovine serum albumin was used for the calibration curve. At least 10 samples (1 sample = 2 guts) from each treatment were used for the analysis.

### 16S rRNA metagenomics

At 48 h posttreatment, the larvae were treated with 0.05% chlorhexidine. Midguts with contents were isolated, homogenized and frozen in liquid nitrogen (4 samples per treatment, 5 midguts per sample). In addition, potato foliage was frozen (3 samples). DNA was isolated using the DNeasy PowerSoil Kit (Qiagen). Bead beating was performed using TissueLyser II (Qiagen) for 10 min at 30 Hz. The V3–V4 region of the 16S rRNA genes was amplified with the primer pair 343F and 806R as described previously [52]. The 16S libraries were sequenced with 2 x 300 bp paired-ends reagents on MiSeq (Illumina) in the SB RAS Genomics Core Facility (ICBFM SB RAS, Novosibirsk, Russia). The read data were deposited in GenBank under the study accession PRJNA699147.

Raw sequences were analyzed with the UPARSE pipeline [53] using Usearch v11.0.667. The UPARSE pipeline included merging of paired reads, read quality filtering, length trimming, merging of identical reads (dereplication), discarding singleton reads, removing chimeras and operational taxonomic units (OTUs) clustering using the UPARSE algorithm [54]. The OTU sequences were assigned a taxonomy using SINTAX [55] and the 16S RDP training set v16 as a reference [56]. The OTUs related to chloroplast 16S rDNA were removed.

The final data set included 507752 reads (reads of midgut: 31734 ± 2440 per sample; reads of foliage: 2289 ± 173) (S1 Appendix). Rarefaction and extrapolated curves were generated using the “iNEXT” package [57] and tended to approach the saturation plateau, which indicated a sufficient number of reads (S1 Fig). Alpha diversity metrics were calculated in Usearch.

### Enterobacteria CFU count and identification

At 48 h posttreatment, the larvae were cryoanesthesiated at -4°C and surface sterilized with 0.05% chlorhexidine. Then, the guts with the contents were isolated, homogenized and suspended in sterile 1 mL 150 mM NaCl (pH 7.2) buffer (3 guts per sample). Aliquots (100 μL) from suspensions diluted at 10^−2^, 10^−3^ and 10^−4^ were inoculated on the surfaces of Endo agar (M029; Himedia, Mumbai, India) and Serratia differential medium (M1288; Himedia, Mumbai, India). Colonies were counted after 48 h of incubation at 28°C. Five samples of each treatment were used for analysis (1 sample = 3 guts). Colonies with typical morphology were isolated into a pure culture by passaging three times on tryptosis agar (M538; Himedia, Mumbai, India) under the same conditions. Identification of pure cultures of 1718 *Enterobacter* sp., 6918 *Citrobacter* sp. and 10918 *Serratia* sp. was carried out using 16S rRNA sequencing. Bacterial cells were suspended in sterile water, heated to 95°C for 5 min, and pelleted by centrifugation. The obtained supernatant was used as a template for the next identification step. Sequencing of the 1308 bp fragment of the 16S rRNA gene was carried out using primers 8F 5’-AGRGTTTGATCCTGGCTCA-3’ and 1350 R 5’-GACGGGCGGTGTGTACAAG-3’. Sanger reaction products were cleaned and subsequently analyzed on an ABI 3130XL automatic gene analyzer (ABI, USA) at the Genomics Core Facility SB RAS. The obtained sequences were deposited in the GenBank database under numbers MW045456, MW045457 and MW045458. The obtained reference sequences from GenBank (http://www.ncbi.nlm.nih.gov) were aligned in BioEdit.

### Antagonistic interaction between *Bt* and culturable Enterobacteriaceae and effect of avermectins on the bacteria

The antagonistic interaction between culturable gut Enterobacteriaceae and *Bt* was studied by the method of double culture on tryptosis agar in 90 mm Petri dishes. Ten mm plugs with freshly plated *Bt* were placed on freshly plated lawns of *Citrobacter*, *Serratia* and *Enterobacter*. Similarly, plugs of *Citrobacter*, *Serratia* and *Enterobacter* were placed on freshly plated lawns of *Bt*. The effect of avermectins on the growth of *Citrobacter*, *Serratia*, and *Enterobacter* was assessed by plating 10 mm paper disks soaked in an avermectin solution (9 μg/L) on freshly plated bacterial culture lawns. Petri dishes were incubated at 28°C. The inhibition zones were measured after 48 h of incubation. Assays were performed with nine replicates.

### Influence of culturable bacteria on the susceptibility of larvae to *Bt* and avermectins

Prior to treatments with Enterobacteriaceae species and their combination with *Bt* or avermectins, the larvae were exposed to the broad-spectrum antibiotic amikacin (JSC Sintez, Kurgan, Russia). The antibiotic at a concentration of 30 mg/L was applied by spraying potato leaves fed to the larvae for 24 h. Inhibition of the bacteria *Enterobacter*, *Citrobacter*, *Serratia* and *Bt* with the antibiotic was estimated *in vitro* using filter paper discs soaked in amikacin at the concentrations mentioned above and placed on freshly plated bacteria cultures. Inhibition zones were measured at 24 h after plating. In addition, the efficacy of enterobacterial eradication was estimated in vivo by plating CPB midguts on Endo agar at 24 h postfeeding with potato leaves that were treated with the antibiotic at the same concentration.

For cotreatments of CPB larvae with enterobacteria and *Bt* or enterobacteria and avermectins, one-day-old cultures of *Enterobacter*, *Citrobacter* and *Serratia* were collected from the surface of the tryptosis agar with a sterile microbiological loop and transferred into sterile 150 mM NaCl. The bacterial suspensions were washed twice in 150 mM NaCl (6000 g, 10 min) and diluted to a final concentration of 5 × 10^8^ cells/mL. The *Bt* suspension and the avermectin solution were prepared as described in the section "Insects, bacteria and insecticides". The final concentrations of *Bt* and avermectins were 1 × 10^8^ spores/mL and 2.25 μg/L, respectively. The surface of the potato leaves was treated with *Bt*, *Enterobacter*, *Citrobacter*, *Serratia*, and *Bt+Enterobacter*, *Bt+Citrobacter*, *Bt+Serratia*, as well as with avermectins, avermectins*+Enterobacter*, avermectins*+Citrobacter*, avermectins*+Serratia* and NaCl as a control. Treated leaves were offered to CPB larvae for 48 h, and then replaced by untreated leaves. The insects were incubated as described above. Mortality was assessed for 6 days. Four replicates were used in each treatment (1 replicate = 10 individuals).

### Statistical analysis

Differences in mortality rate were analyzed using the Kaplan-Meier log-rank test followed by a Holm-Sidak adjustment (Sigma-Stat 3, Systat Software Inc, USA). The synergistic and additive effects on CPB mortality were determined by comparing the expected and observed mortality using the χ^2^ criterion, as described by Robertson and Preisler [58]. Data on the enzyme activity, OTU abundance, diversity indexes, CFU count and antagonistic activity of culturable bacteria and the agents were checked for normal distribution using the Shapiro-Wilk W test. The normally distributed data were analyzed using two-way ANOVA followed by Tukey’s post hoc test (STATISTICA 8, StatSoft Inc., USA). For the non-normal distribution, the nonparametric analog of two-way ANOVA, the Scheirer-Ray-Hare test [59], was used, followed by Dunn’s post hoc test. Differences in the antagonistic effect of *Bt* against gut symbiotic bacteria were analyzed by one-way ANOVA followed by Tukey’s post hoc test.

## Results

### Additive effect between *Bt* and avermectins in CPB mortality

**Fig 1:**
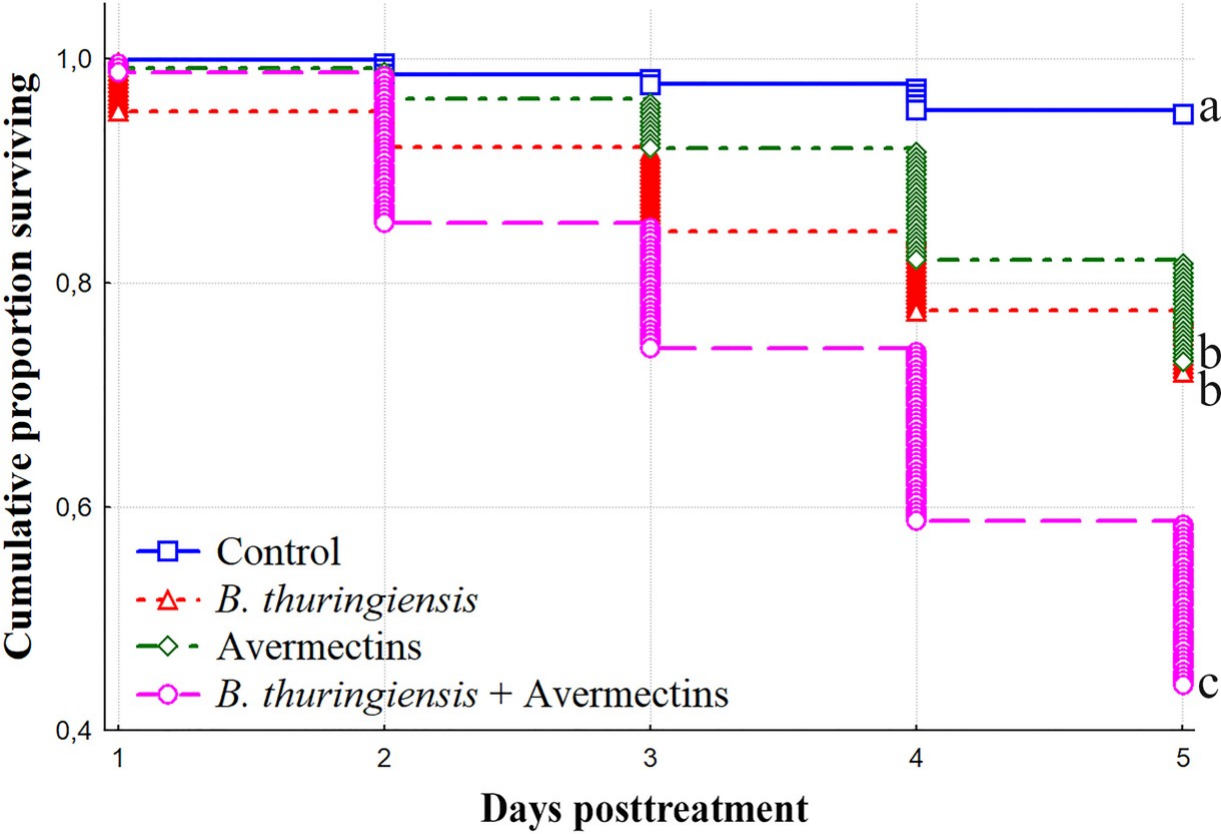
The mortality dynamics of the CPB IV-instar larvae after treatments with *Bacillus thuringiensis* (*Bt*) (3 × 10^7^ spores/mL), avermectins (1.125 μg/L) and their combination. Different letters (a-c) indicate significant differences between treatments estimated by the log rank test (χ^2^ > 38.5, df = 1, p < 0.001).

We used potato foliar assay to evaluate the effect of ingestion of *Bt* or avermectin by CPB larvae. Feeding the larvae with potato leaves treated with *Bt* (3 × 10^7^ spores/mL) or avermectins (1.125 μg/L) led to 28.4 and 27.2% mortality on the 5^th^ day posttreatment, respectively, which significantly differed from the control group of insects (log rank test, χ^2^ > 39.9, df = 1, p < 0.001, Fig 1). The treatment with the combination of *Bt* and avermectins increased the mortality of the larvae, reaching 59.7% on the 5^th^ day posttreatment, which significantly differed from the single treatments (χ^2^ > 38.5, df = 1, p < 0.001). However, only an additive effect between *Bt* and avermectins was observed from the 1^st^ to 5^th^ days posttreatment (χ^2^ < 3.5, df = 1, p > 0.05). The mortality of control larvae reached 5.1% on the 5^th^ day posttreatment.

### Electron microscopy of the midgut

**Fig 2:**
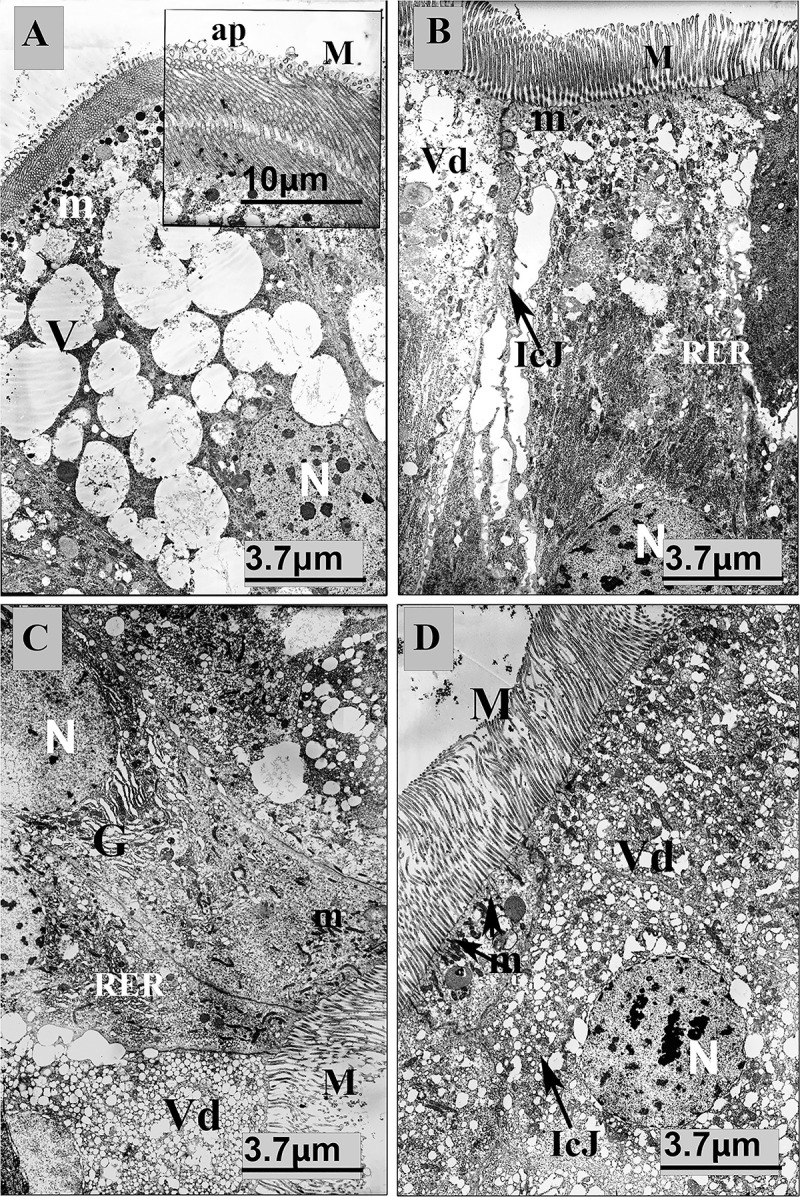
Ultrathin sections of the IV-instar CPB larvae midgut at 48 h posttreatment with *B*. *thuringiensis* (*Bt*) (3 × 10^7^ spores/mL), avermectins (1.125 μg/L) and their combination. (A) Control samples; (B) Treatment with *Bt*; (C) Treatment with avermectins; (D) Combined treatment (*Bt*+avermectins). M–microvilli; ap–apocrine bubbles; m–mitochondria; N–nucleus; V–vesicles; RER–rough endoplasmic reticulum; IcJ–intercellular junctions; Vd–vacuolization; G–Golgi apparatus.

One of the reasons for the additive effect in mortality of CPB larvae could be histolysis of the gut tissues as a result of the toxic action of *Bt* and avermectins. To verify this, we conducted a histological examination of ultrathin sections of the CPB midguts. Columnar cells with microvilli, apocrine bubbles, mitochondria, nuclei, vesicles, and rough endoplasmic reticulum were clearly seen on a gut section of the control sample (Fig 2A). Bacterial treatment (Fig 2B) caused a partial depletion of the cytoplasm and RER condensation. These changes were accompanied by partial loss of secretory vesicles and weakly prominent destructive changes in intercellular junctions. Treatment with avermectins (Fig 2C) induced pronounced destructive effects in the columnar cells, such as (i) the depletion of the cytoplasm, (ii) vacuolization of the intracellular space and (iii) partial destruction of the microvilli. In the perinuclear area, vacuolated areas of the Golgi apparatus were visible. However, the nucleus and mitochondria showed no morphological changes compared to the control samples. The combined treatment with *Bt* and avermectins (Fig 2D) led to the same effect as the treatment with avermectins alone.

### Alpha-amylase and total alkaline protease activity

**Fig 3:**
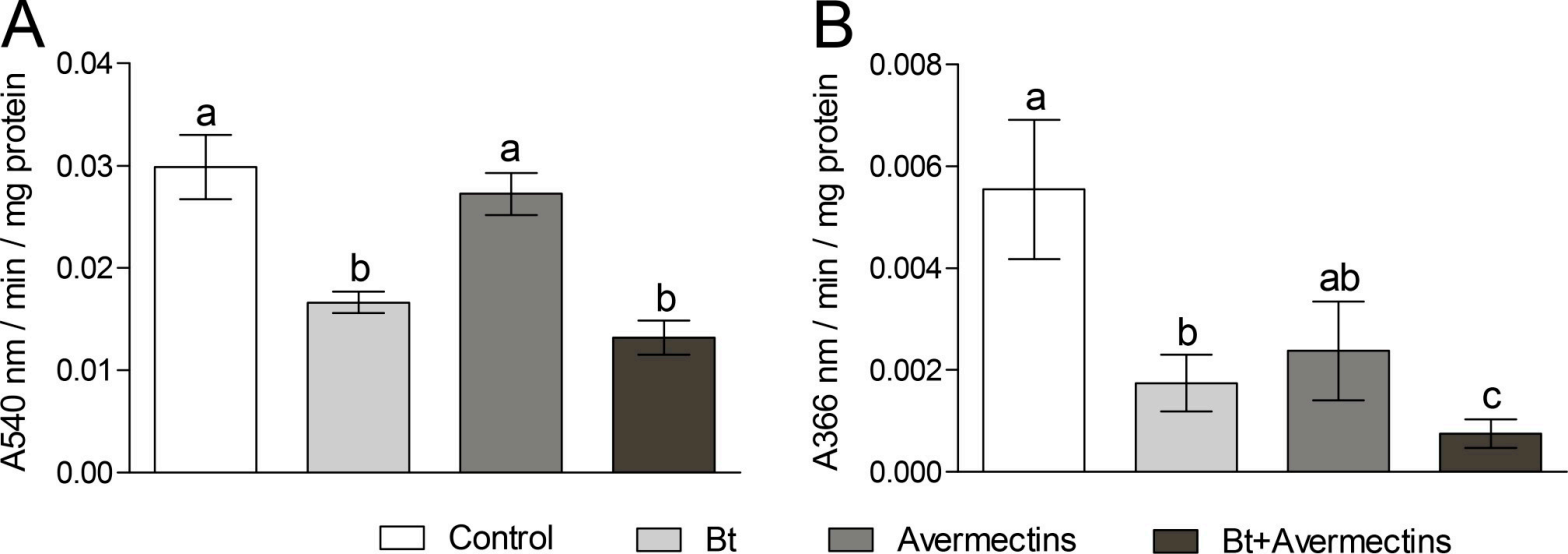
α-Amylase (A) and total alkaline proteolytic activity (B) in the CPB larvae midgut at 48 h posttreatment with *Bacillus thuringiensis* (*Bt*) (3 × 10^7^ spores/mL), avermectins (1.125 μg/L) and their combination. The different letters above the columns indicate significant differences between treatments (Tukey’s test, p < 0.01, for α-amylase activity and Dunn’s test, p < 0.05, for total alkaline proteolytic activity).

Since the destructive effects of the toxicoses on gut tissues could change the activity of digestive enzymes in CPB larvae, we measured the activity of alpha-amylases and total alkaline proteases. Forty-eight hours posttreatment, we observed a significant (1.8–2.1-fold) decrease in α-amylase activity under the influence of *Bt* (two-way ANOVA, F_1.53_ = 42.0, p < 0.001, Fig 3A). The effect of avermectins, as well as the interaction between the two factors, on α-amylase activity was not significant (F_1.53_ = 2.1, p = 0.16 and F_1.53_ = 0.04, p = 0.85, respectively).

The activity of alkaline proteases was characterized by a different pattern (Fig 3B). Treatments with *Bt* and avermectins resulted in a decrease in the enzyme activity, among which treatment with *Bt* led to a more pronounced effect (Scheirer-Ray-Hare test, effect of *Bt*, H_1.31_ = 7.4, p = 0.007; effect of avermectins, H_1.31_ = 4.5, p = 0.034). The two-way analysis (Scheirer-Ray-Hare test) did not reveal significant interactions between *Bt* and avermectins (H_1.31_ = 0.28, p = 0.60), although the strongest decrease was observed after the combined treatment (Dunn’s test, p < 0.05 compared to other treatments).

### Changes in the structure of the midgut bacterial community of CPB

**Fig 4:**
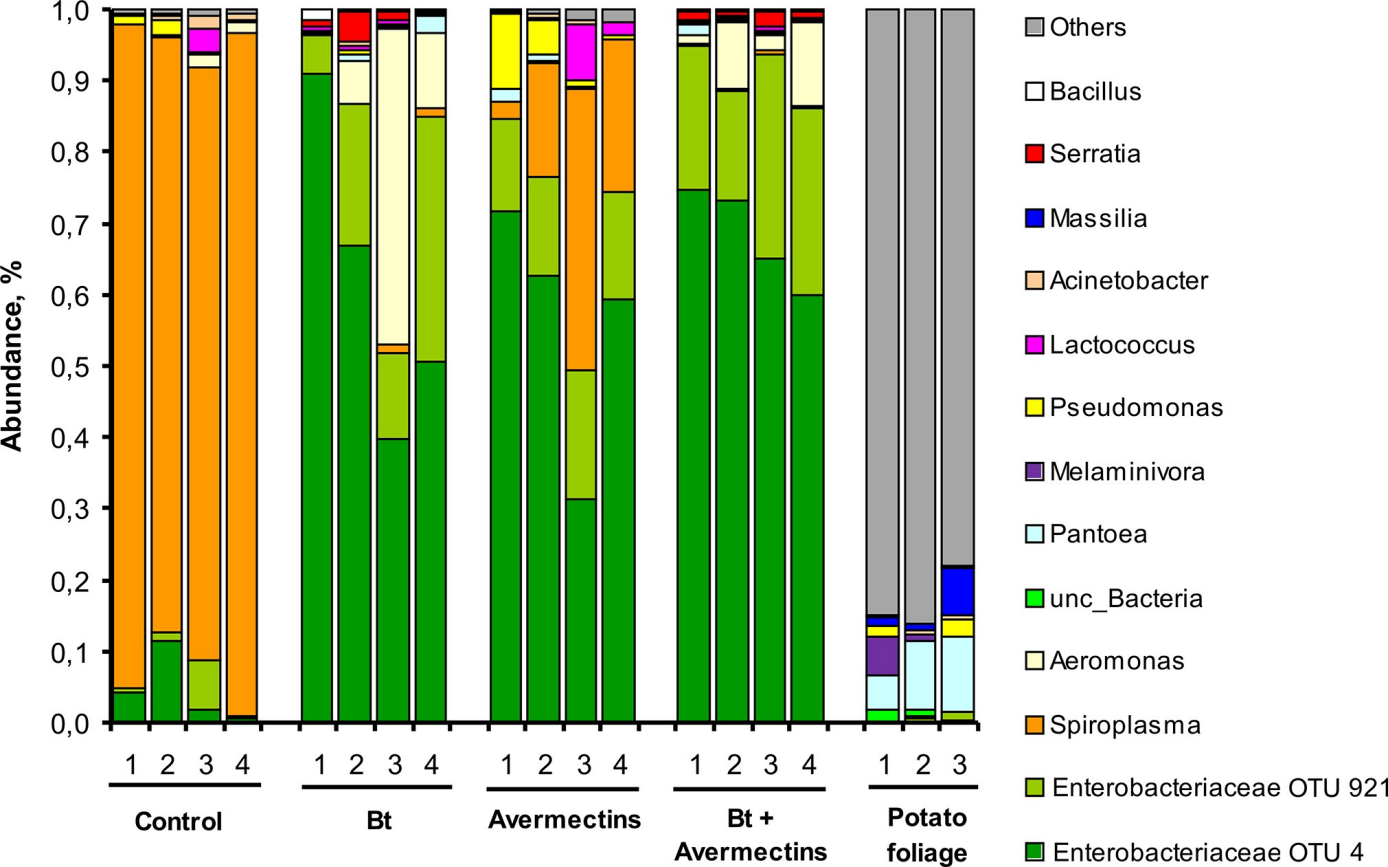
Changes in the structure of the midgut microbiome (genus level) of CPB larvae at 48 h posttreatment with *B*. *thuringiensis* (*Bt*) (3 × 10^7^ spores/mL), avermectins (1.125 μg/L) and their combination. Each treatment presented four replicates, and the potato leaves presented three replicates.

To identify possible changes in the bacterial community under the influence of *Bt* and avermectins, we performed 16S rRNA metagenomics sequencing of CPB larvae midguts. We identified 105 OTUs in the midgut of CPB larvae, which included 50 families grouped into 16 classes (S1 Appendix). The most abundant were Enterobacteriaceae (class Gammaproteobacteria) and Spiroplasmataceae (class Mollicutes) (Fig 4). Enterobacteriaceae were presented by two predominant OTUs, among which OTU4 was close to the different type strains of *Kluyvera*, *Enterobacter*, *Klebsiella* and *Citrobacter* (identity of 99.77–100%), and OTU921 showed only 99.07% identity with the type strains of *Leclercia*, *Enterobacter* and *Morganella*. Spiroplasmataceae were represented by only one OTU with a 100% identity with *Spiroplasma leptinotarsae* only. In addition, one other predominant taxon, unclassified *Aeromonas*, was represented by one OTU that was close to *Ae*. *rivipollensis* (100% identity) and *Ae*. *veronii* (99.77% identity). To clarify contribution of plant bacteria on the microbiome of CPB, we also analyzed the bacterial community of potato foliage. The potato foliage samples included 86 OTUs, which were presented predominantly by Gammaproteobacteria, Betaproteobacteria, Bacilli and Actinobacteria. The Enterobacteriaceae, represented by OTU4 (Enterobacteriaceae), OTU921 (Enterobacteriaceae), and OTU97 (*Pantoea* sp.), were found to be common in the CPB gut and leaves (Fig 4). However, these OTUs had low abundance in the foliage. Therefore, microbiota of CPB larvae was specific, i.e. significantly differed from potato foliage.

Treatments with *Bt* and avermectins led to significant changes in the structure of the gut bacterial community (Fig 4). Control insects were characterized by a predominance of *S*. *leptinotarsae* in the midgut (82–95%). Under the influence of *Bt* and avermectins, a significant decrease in the relative abundance of *S*. *leptinotarsae* was observed, and the strongest effect was documented after *Bt* treatment (effect of *Bt*: F_1.12_ = 168.24, p < 0.0001; effect of avermectins: F_1.12_ = 69.60, p < 0.0001). At the same time, treatments with both agents alone or their combination led to a significant increase in the relative abundance of Enterobacteriaceae OTU4 and OTU921 (effect of *Bt*: F_1.12_ > 10.52, p < 0.007; effect of avermectins: F_1.12_ > 5.89, p < 0.032). In addition, under the influence of *Bt*, a significant increase in the relative abundance of *Serratia* (F_1.12_ = 11.23, p = 0.005, Fig 4) and a trend toward an increase in the abundance of *Aeromonas* (F_1.12_ = 4.01, p = 0.07) were observed. The relative abundance of *Bacillus* in the midguts of insects treated with *Bt* alone was 0.34 ± 0.31%, which was 94-fold higher than that in control larvae (Dunn’s test, p = 0.03). At the same time, *Bacillus* abundance was zero in the midguts of larvae treated with avermectins. The abundance of *Bacillus* in the midguts of larvae treated with a combination of *Bt* and avermectins was similar to that in the control (0.006% and 0.004%, respectively, Dunn’s test, p = 0.62).

To quantify shifts in the diversity of bacterial communities under the influence of avermectins and *Bt*, we calculated Chao1 and Shannon diversity indices. Chao1 reflects potential diversity and Shannon index is sensitive to equability in abundances of taxa in a sample. *Bt* and avermectin treatments led to a decrease in the number of OTUs and Chao1 index; however, these effects were not significant (Scheirer-Ray-Hare test, effect of avermectins: H_1.12_ < 2.31, p > 0.12; effect of *Bt*: H_1.12_ < 0.27, p > 0.59) (Table 1). However, treatment with avermectins alone led to a significant decrease in these indexes compared to the control (Dunn’s test, p < 0.02). *Bt* and avermectins single treatments significantly increased the Shannon index (Dunn’s test, p < 0.02, compared to the control). However, combined treatment led to a slight and insignificant increase in the index (Dunn’s test, p = 0.21, compared to control).

**Table 1:** Diversity characteristics of bacterial communities in the midgut of CPB larvae 48 h posttreatment with *B*. *thuringiensis*, avermectins and their combinations.

Treatments	Diversity indexes		
	OTUs	Shannon	Chao1
Control	39 ± 3.63^a^	0.52 ± 0.12^a^	42.5 ± 3.75^a^
*B*. *thuringiensis*	27 ± 4.9^ab^	0.99 ± 0.19^b^	30.55 ± 6.3^ab^
Avermectins	21 ± 2.25^b^	1.17 ± 0.09^b^	24.04 ± 3.22^b^
*B*. *thuringiensis +* Avermectins	27 ± 2.93^ab^	0.92 ± 0.05^ab^	30.52 ± 4.88^ab^

The standard errors (±SE) were calculated for 4 replicates. Different letters indicate significant differences among treatments (Dunn’s post hoc test, p < 0.05).

### Changes in CFU counts

**Fig 5:**
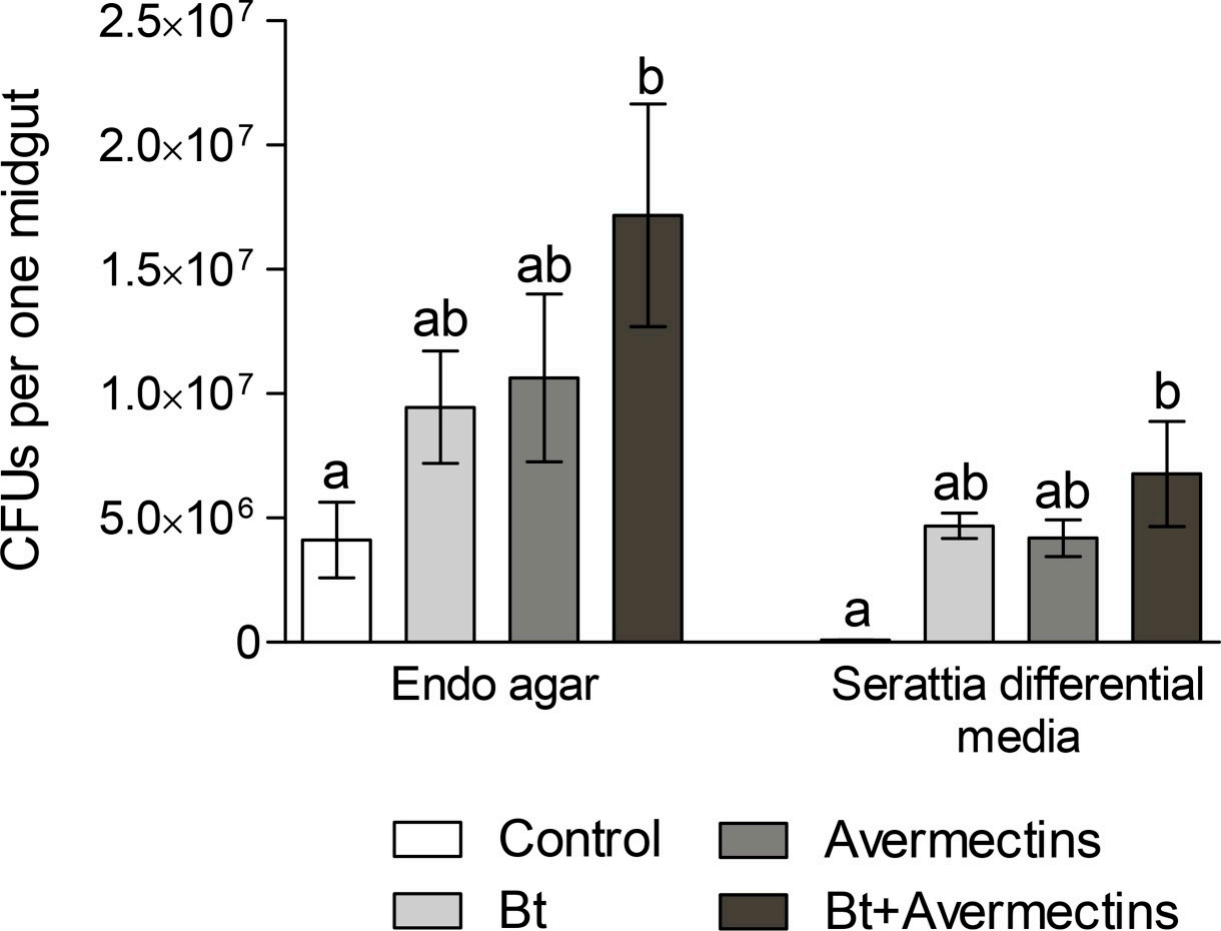
Changes in CFU counts of Enterobacteriaceae in the CPB larvae midgut at 48 h posttreatments with *B*. *thuringiensis* (*Bt*) (3 × 10^7^ spores/mL), avermectins (1.125 μg/L) and their combination. The letters above the columns indicate significant differences (Tukey’s test, p < 0.05, compared to the control).

To confirm the increase of Enterobacteriaceae in the gut of CPB larvae after oral administration to *Bt* and avermectins, we performed analysis of CFU count. Inoculation of the gut homogenates of CBP larvae on Endo agar revealed a significant increase in the CFUs of enterobacteria under the influence of avermectins (F_1.16_ = 5.2, p = 0.04, Fig 5). *Bt* treatments also led to an increase in CFU count, but the effect was at the marginal significance level (F_1.16_ = 3.6, p = 0.07). Post hoc tests showed that single treatments resulted in a 2.3–2.5-fold (but insignificant) increase in CFU count (Tukey’s test, p > 0.4, compared to control), while combined treatment led to a 4.2-fold significant increase in CFU count (Tukey’s test, p = 0.04 compared to control). A similar pattern has been observed for CFUs on the Serratia differential medium. Under the influence of both agents, an increase in CFU count occurred (effect of *Bt*: F_1.15_ = 8.8, p = 0.01; effect of avermecrins: F_1.15_ = 6.5, p = 0.02) (Fig 5). The post hoc test showed significant differences only between the combined treatment and the control (Tukey’s test, p = 0.006), while single treatments led only to the trend toward an increase in CFU count (p > 0.08). No significant factor interactions between avermectins and *Bt* on CFU count on either media were observed (F < 0.7, p > 0.42).

### Identification of enterobacteria and their interaction with *Bt* and avermectins

The predominant bacterial colonies were selected for species identification with subsequent analysis of antagonistic interactions with *Bt* and avermectins *in vitro*. Sequencing of 16S rRNA type colonies cultured on Endo agar and Serratia differential media showed their high similarity with type cultures of *Enterobacter ludwigii* (99.93%), *Citrobacter freundii* (99.92%), and *Serratia marcescens* (99.86%) (Table 2).

**Table 2:** Similarity and coverage of 16S sequences (%) of Enterobacteriaceae isolates using the BLAST algorithm in comparison with similar sequences obtained from GenBank.

Tested strain	Test sequence length, nucleotide pairs	Coverage, %	Identities, %	Nearest matches (GenBank Ids)
1718	1303	100	99.93	*Enterobacter ludwigii* strain EN-119 (NR_042349.1)
6918	1274	100	99.92	*Citrobacter freundii* ATCC 8090 (MTCC 1658 strain LMG 3246)
10918	1424	100	99.86	*Serratia marcescens* strain NBRC 102204 (NR_114043.1)

**Fig 6:**
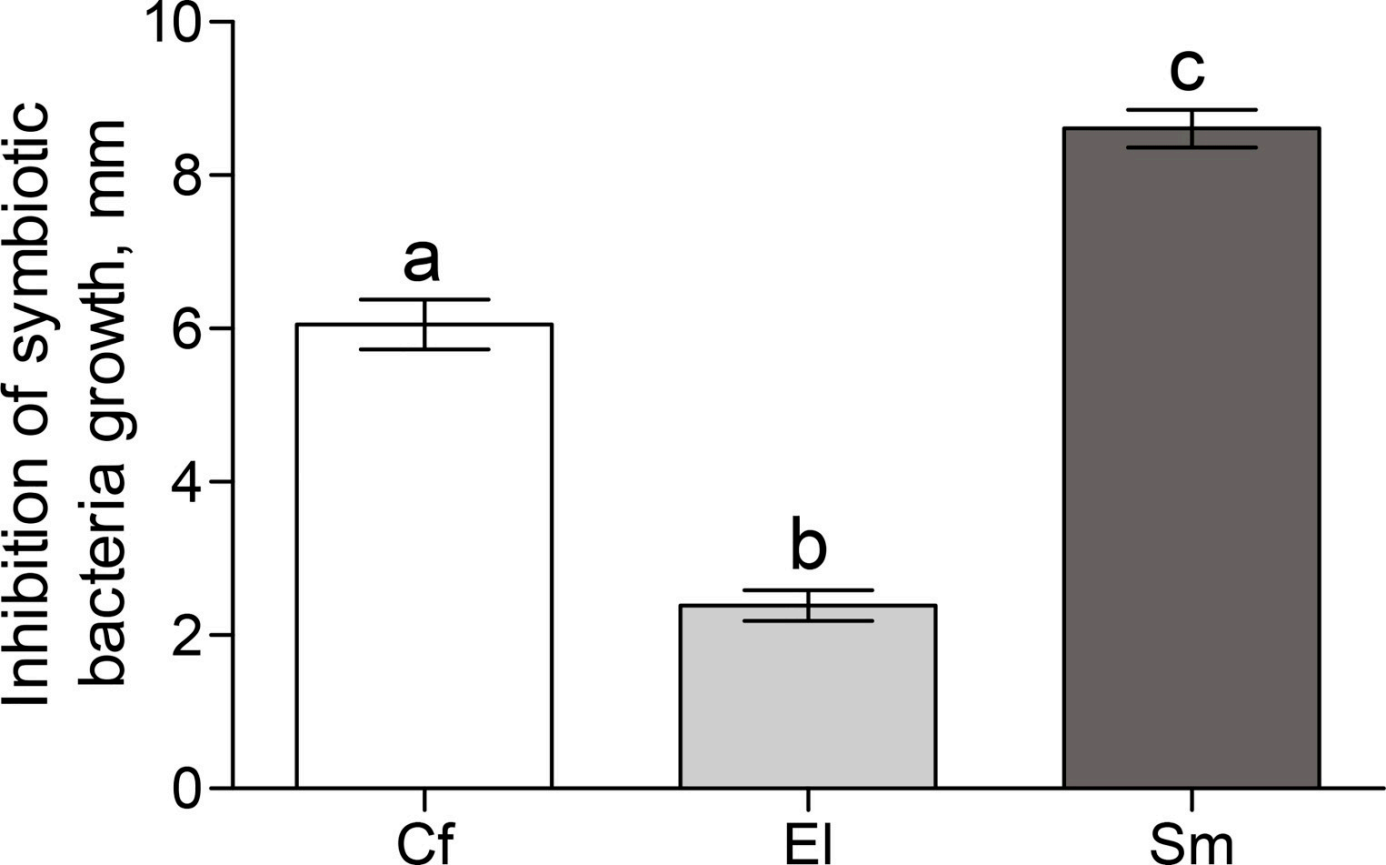
Antagonistic effect of *B*. *thuringiensis* against gut symbiotic bacteria of CPB larvae assessed by the method of double culture. Cf–*Citrobacter freundii*, El–*Enterobacter ludwigii*, Sm—*Serratia marcescens*. Different letters above the columns show the significance of the differences between treatments (Tukey’s test, p < 0.001).

Analysis of antagonistic interactions between *C*. *freundii*, *E*. *ludwigii*, *S*. *marcescens* and *Bt* using the agar plug method showed that these bacteria did not inhibit *Bt* growth. In contrast, *Bt* inhibited the growth of cultured gut bacteria (Fig 6). The weakest antagonism was observed against *E*. *ludwigii*, in which the zone of inhibition did not exceed 2.3 ± 0.2 mm. *Bt* had a significant inhibitory effect against cultures of *C*. *freundii* and *S*. *marcescens*, and the inhibition zones were 6.0 ± 0.3 mm and 8.6 ± 0.2 mm, respectively. We did not find any inhibitory effects of avermectins at the concentrations used on the growth of *C*. *freundii*, *E*. *ludwigii*, *S*. *marcescens* and *Bt*.

### Influence of symbiotic bacteria on CPB susceptibility to *Bt* and avermectins

Prior to assaying the influence of the symbiotic bacteria on CPB susceptibility to *Bt* and avermectins, we estimated the influence of amikacin on the bacteria. All investigated bacteria were sensitive to amikacin *in vitro* (S2 Fig), and applying the antibiotic to foliage led to a strong decrease in the Enterobacteriaceae CFU count in the CPB midgut 24 h posttreatment (S3 Fig).

**Fig 7:**
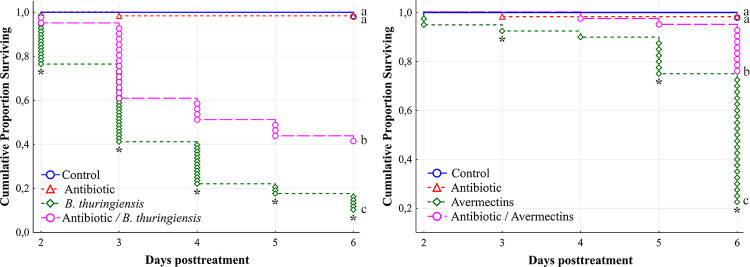
Mortality dynamics of native CPB larvae pretreated with antibiotic (amikacin; 30 mg/L) after treatment with *B*. *thuringiensis* (1 × 10^8^ spores/mL) and avermectins (2.25 μg/L). Different letters (a-с) indicate significant differences between treatments determined by the log rank test (χ^2^ > 12.4, df = 1, p < 0.005). Asterisks indicate antagonistic effects (χ^2^ > 3.84, df = 1, p < 0.05).

Larvae pretreated with amikacin were more resistant to both *Bt* (1 × 10^8^ spores/mL) and avermectins (2.25 μg/L) (Fig 7). In particular, significant differences in susceptibility to *Bt* between native and amikacin-treated insects were observed (log rank test, χ^2^ = 13.9, df = 1, p < 0.001), and an antagonistic effect between antibiotic pretreatment and *Bt* infection was revealed from the 2^nd^ to 6^th^ days postinfection (χ^2^ > 6.5, p < 0.02). Similarly, avermectins treatment led to significant differences in the mortality dynamics of native larvae and larvae pretreated with amikacin (χ^2^ = 12.4, df = 1, p < 0.001), and antagonism between the antibiotic and avermectins was documented on the 3^rd^, 5^th^ and 6^th^ days posttreatment with avermectins (χ^2^ > 4.1, p < 0.05).

**Fig 8:**
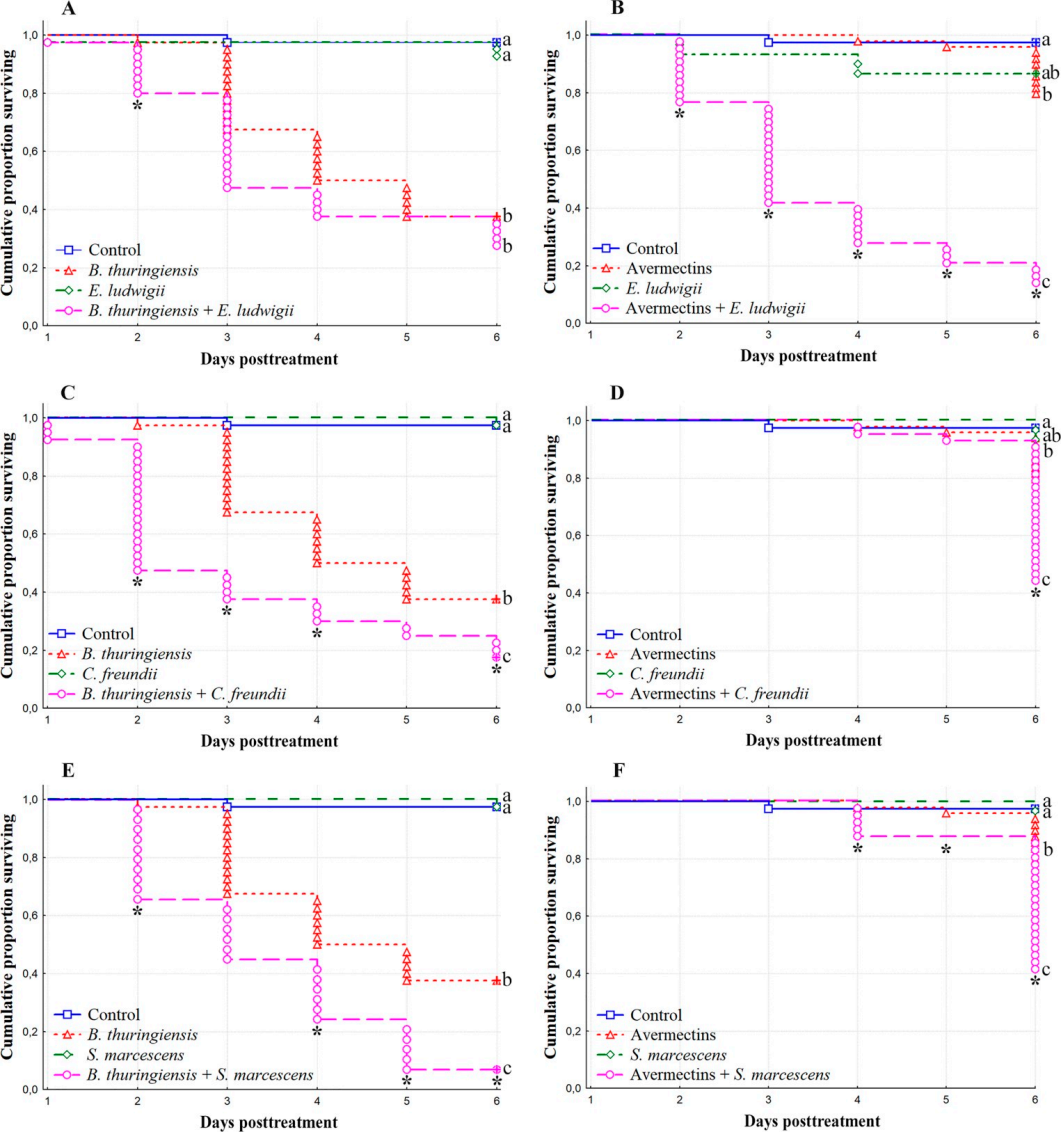
The mortality dynamics of the CPB larvae midgut after treatments with (A, C, E) *B*. *thuringiensis* (*Bt*) (1 × 10^8^ spores/mL), (B, D, F) avermectins (2.25 μg/L) and reintroduction of symbiotic bacteria (5 × 10^8^ cells/mL). (A, B)–*Enterobacter ludwigii*; (C, D)–*Citrobacter freundii;* (E, F)–*Serratia marcescens*. Different letters (a-с) indicate significant differences between treatments determined by the log rank test (χ^2^ > 7.9, df = 1, p < 0.01). Asterisks indicate synergistic effects (χ^2^ > 3.84, df = 1. p < 0.05).

At the second stage of this work, we analyzed the role of the isolated bacterial strains in the susceptibility of antibiotic-pretreated larvae to *Bt* (1 × 10^8^ spores/mL) and avermectins (2.25 μg/L). All tested bacterial strains (5 × 10^8^ cells/mL) did not affect larval mortality compared to the control (Fig 8). However, when combined with *Bt*, additive or synergistic effects were observed. The reintroduction of *E*. *ludwigii* led to a slight increase in larval mortality due to *Bt*. Differences in mortality dynamics after treatment with *Bt* and *Bt*+*E*. *ludwigii* were not significant (log rank test, χ^2^ = 2.1, df = 1, p = 0.27, Fig 8A), and the effect was additive from 3^rd^ to 6^th^ days posttreatment (χ^2^ < 1.7, p > 0.05). *C*. *freundii* led to a significant increase in susceptibility to *Bt*. The median lethal time decreased 2.5-fold after the combined treatment compared to treatment with *Bt* alone (log rank test, χ^2^ = 7.9, df = 1, p = 0.01) (Fig 8C), and synergy from the 2^nd^ to 4^th^ and on the 6^th^ day posttreatment was observed (χ^2^ > 4.6, p < 0.05). Treating the larvae with *S*. *marcescens* also increased susceptibility to *Bt*. The median lethal time decreased 1.3-fold after combined treatment compared to infection with *Bt* alone (log rank test, χ^2^ = 11.2, df = 1, p < 0.001) (Fig 8E), and a significant synergistic effect was observed on the 2^nd^ and the 4^th^ to 6^th^ days posttreatment (χ^2^ > 10.0, p < 0.01) (Fig 8E).

Combinations of all tested gut bacteria with avermectins led to an increase in mortality compared to treatment with avermectins alone (log rank test, χ^2^ > 9.1, df = 1, p < 0.008) and resulted in a synergistic effect in all treatments (Fig 8). In particular, *E*. *ludwigii* increased mortality due to avermectins as soon as the second day posttreatment, and synergy was documented until the 6^th^ day of the experiment (χ^2^ > 22.0, p < 0.001) (Fig 8B). The reintroduction of *C*. *freundii* led to a synergistic effect that was recorded only on the 6^th^ day posttreatment (χ^2^ = 17, p < 0.001) (Fig 8D). Cotreatment with avermectins and *S*. *marcescens* led to a synergistic effect from the 4^th^ to 6^th^ days posttreatment (χ^2^ > 4.7, p < 0.05) (Fig 8F).

## Discussion

In this study, we showed that *B*. *thuringiensis* var. *tenebrionis* (*morrisoni*) and avermectins interact additively in CPB larval mortality under laboratory conditions. We suggest that the increase in mortality is associated with destructive changes in the larval gut tissues that likely affect the activity of some digestive enzymes, and with an increase in the number of enterobacteria under the influence of both agents.

Earlier, a synergistic effect on the mortality of CPB was shown with the combined treatment of larvae with entomopathogenic fungi and insecticides [17, 60, 61], in particular avermectins and *Metarhizium robertsii* [18, 19]. Presumably, the more pronounced effect on larval mortality from fungi when combined with avermectins is due to the different mechanisms of action, which is mainly intestinal for avermectins and cuticular for fungi. At the same time, the effect of increased mortality was weaker under the influence of *Bt* and avermectins with similar (intestinal) modes of action. This is to be expected since both agents significantly slow food consumption by CPB [19, 62]. Therefore, toxicity caused by avermectins can lead to a decrease in the concentration of *Bt* spores and crystals in the gut, and vice versa, a slowdown in nutrition under the influence of *Bt* leads to a decrease in the consumed doses of avermectins. A similar effect was found in mosquito larvae after their simultaneous treatment with avermectins and *M*. *robertsii* conidia, which accumulated in the mosquito guts [63]. In particular, under the influence of avermectins, the CFU count of the fungus in the larvae significantly decreased. However, although both avermectins and *Bt* are able to decrease food (and, accordingly, the agents) consumption, we observed an increase in the mortality of CPB larvae.

It is known that histolysis of gut tissues occurs during *Bt* bacteriosis, as shown on Lepidoptera [27, 64, 65] and some Coleoptera [10]. It was also shown that exposure of *Apis mellifera* and different mosquito species to median lethal doses of avermectins led to gut histolysis [23, 22]. In our study, ultrathin sections of the midgut tissues of CPB larvae 48 h posttreatment with avermectins showed pronounced destructive effects. In particular, depletion and vacuolization of the cytoplasm, tissue decay in the perinuclear space, as demonstrated by the destruction of the Golgi apparatus, intercellular septa and microvilli, were observed in the columnar cells. These disorders in gut cells may be associated with suppression of the functioning of glutamate-gated chloride channels and β-amino acid receptors. Oncosis of the gut cells and peristaltic paralysis have been observed as a result of avermectin treatment [66, 67]. We assume that the violation of the integrity of the gut cells facilitates the penetration of *Bt* and resident bacteria into the hemocoel. It is known that minor damage to gut epithelial cells when exposed to sublethal doses of *Bt* can lead to slow necrosis but can be restored by strengthening the immune response [68]. However, avermectins lead to more pronounced destructive tissue changes that may disrupt the restoration of epithelial cells and facilitate the penetration of *Bt* and resident bacteria into the insect’s hemocoel.

Another indicator of toxic effects is a violation of the intensity of the digestive processes. Toxicoses are usually accompanied by a decrease in the activity of digestive enzymes. α-Amylases play an important role in the hydrolysis of starch in insects [69, 70], and a decrease in their activity was found in *Spodoptera littoralis* when exposed to half-lethal doses of *Bt* [71]. We also revealed a significant decrease in the activity of α-amylases when CPB larvae were exposed to *Bt* and to a combination of *Bt* with avermectins, which indicates a violation of homeostasis in the gut and the destruction of cells responsible for the secretion of enzymes during bacterial toxicosis. Another factor that determines the toxic effect is a change in the activity of proteases in the insect midgut [72]. Alkaline proteases, on the one hand, are involved in the activation of *Bt* toxins, and on the other, are able to destroy them [27, 73, 74]. These enzymes play an important role both in the defensive responses and in digestion processes, and any change in their activity or in the composition of intestinal proteolytic enzymes can affect insect sensitivity to insecticides and entomopathogens [75, 76]. In our study, the combined action of *Bt* and avermectins led to the strong suppression of the activity of alkaline proteolytic enzymes in the larval gut, which could probably contribute to an increase in CPB mortality.

The results of the metagenomic analysis of the 16S rRNA V3–V4 regions revealed that the most common gut symbionts of CPB were spiroplasmas and enterobacteria. Native insects had a very high relative abundance of the obligate symbiont *S*. *leptinotarsae* in the gut (up to 95%). In our previous studies on the same population of CPB [46, 61], relative abundance of this bacteria was lower (40–53% in untreated larvae). Huckett and coworkers have previously shown [77] that the occurrence of *S*. *leptinotarsae* in CPB varies considerably, which may be influenced by many factors, including climatic condition, CPB population density and laboratory rearing. We observed a significant decrease in the relative abundance of *S*. *leptinotarsae* simultaneously with a sharp increase in the abundance of Enterobacteriaceae after the treatments with *Bt* or avermectins. A decrease in the abundance of *S*. *leptinotarsae* under the influence of *Bt* and avermectins is predictable, since Spiroplasma is an intracellular obligate symbiont [78] and the destruction of the gut epithelium may lead to its elimination. A simultaneous sharp increase in the abundance of Enterobacteriaceae may be associated with destructive processes in the gut tissues, slowing down peristalsis and the movement of food through the gut, creating favorable conditions for these bacteria since they are facultative anaerobes. An increase in the abundance of Enterobacteriaceae in insects under various pathological effects associated with malnutrition is consistent with other studies. For example, Dubovskiy *et al*. [30] showed an increase in Enterobacteriaceae during *Bt* infection in the wax moth. The neurotoxin of the parasitoid *Habrobracon hebetor*, which causes the arrest of gut peristaltic activity, also leads to the proliferation of enterobacteria in the wax moth [79]. The increase in the abundance of Enterobacteriaceae under the influence of *Bt* and avermectins was confirmed by CFU analysis, while the strongest and most significant increase occurred under the combined action of these agents.

The identified enterobacteria belonged to the species *E*. *ludwigii*, *C*. *freindii*, and *S*. *marcescens*. Previously, we established that these bacteria are the dominant representatives of the native flora in the studied beetle population (Kryukov, Polenogova, and Krivopalov, unpublished). Pretreatment of insects with an antibiotic that inhibits the growth of these bacteria resulted in increased larval resistance to both *Bt* and avermectins (Fig 7). In addition, oral administration of these bacteria to the larvae led to increased mortality due to *Bt* infection at the additive or synergistic levels. Earlier, it was shown that enterobacteria significantly increase the virulence of *Bt* in the Lepidoptera *Lymantria dispar* [37]. Damage of the gut caused by *Bt* toxin accelerates the penetration of bacterial associates into the hemocoel, which accelerates the death of lepidopterans due to *Bt* [38, 40]. Despite the strong antagonism of *Bt* against enterobacteria *in vitro*, these residents probably contribute to the accelerated development of septicemia in the CPB larvae infected with *Bt*.

We have also shown that Enterobacteriaceae increase the susceptibility to avermectins. Insects treated with the antibiotic were less susceptible to avermectins, but reintroduction of enterobacteria resulted in increased susceptibility of the larvae to avermectins at the synergistic level. The strongest synergistic agent was *E*. *ludwigii*. A number of studies have shown opposite effects in interactions between enterobacteria and insecticides. For example, *Citrobacter* sp., a gut symbiont of the fruit fly *Bactrocera dorsalis*, is able to degrade the organophosphorus insecticide trichlorphon [44]. Flies inoculated with the bacteria showed increased resistance to trichlorphon compared to antibiotic-treated flies. Hernández-Martínez *et al*. [65] demonstrated that a reduced load of enterobacteria led to the increased susceptibility of *Spodoptera exigua* to compounds from *Azadirachta indica*. Wang and co-workers [42] showed that microbiome-mediated resistance to atrazin in the wasp *Nasonia vitripennis* is mediated by *S*. *marcescens*, which participates in the degradation of this insecticide. Gomes and coauthors [80] established that many enterobacteria (*Enterococcus*, *Klebsiella*, *Leclercia*) from the *Spodoptera frugiperda* gut metabolize different insecticides, such as flubendiamide, indoxacard, chlorantraniliprole, lufenuron, teflubenzuron and spinosad, as carbon sources. However, other authors have shown an increase in susceptibility to insecticides under the influence of enterobacteria. For example, Xia *et al*. [34] demonstrated that inoculation of *Plutella xylostella* with *S*. *marcescens* resulted in decreased resistance to chlorpyrifos. To our knowledge, there are no studies on the effects of Enterobacteriaceae on avermectins, although interactions have been shown for some other bacteria. In particular, Fernandez and co-workers [81] showed that the wasp *Eretmocerus mundus* pretreated with antibiotics was more susceptible to abamectin. The authors suggest that resistance to abamectin is caused by the bacteria *Arthrobacter* (Micrococcaceae), which exhibits esterase activity and may be involved in the degradation of pesticides. It seems that interactions between insecticides and symbiotic bacteria may be unique for each insect-insecticide system and dependent on dosages and host gut physiology. According to our results, we support the idea that the main mechanism of these interactions is not the direct detoxification of insecticides by gut bacteria [34]. After damage to gut tissues by avermectins, enterobacteria can show pathogenic properties faster than detoxifying properties. Therefore, we observed a high susceptibility to avermectins in the CPB larvae harboring enterobacteria. Most likely, additional damage to gut tissues by *Bt* may increase the pathogenic properties of the gut bacteria and lead to an additive effect between *Bt* and avermectins.

It should be noted that the development of septicemia caused by intestinal residents under the influence of *Bt* depends on the level of cellular immunity of the insect [38]. Previously, we demonstrated a decrease in the parameters of cellular immunity under the influence of avermectins in CPB larvae. Namely, there is an increase in necrosis and apoptosis of hemocytes and a decrease in the number of granulocytes [18]. Obviously, these changes may also promote septicemia caused by *Bt* and gut residents. Thus, we show for the first time that bacterial associates stimulate the development of *Bt*-bacteriosis in Coleoptera larvae, particularly in CPB. This was shown previously only in Lepidoptera [31, 37, 38].

In conclusion, we showed that avermectins and *Bt* interact additively when simultaneously applied to the food of CPB larvae. The increase in mortality under the combined treatment is due to destructive changes in the gut tissues of the CBP larvae, changes in the activity of digestive enzymes and an increase in the abundance of Enterobacteriaceae that accelerate both *Bt* infection and avermectins toxicosis. We confirm the hypothesis of the importance of the gut microbiota in the development of *Bt* infections, as previously shown for lepidopterans [31, 37, 38]. Moreover, we demonstrated that enterobacteria increase the susceptibility of CPB larvae to avermectins. Importantly, this study shows that the additive effects on insect mortality under the combined treatment of insecticides and pathogens are not only due to the direct physiological responses of insects to these agents but can also be associated with changes in the abundance of insect bacterial associates. The revealed combination can be used for developing complex products for biological control of the CPB population. Subsequent studies can be aimed at testing this combination in the field and developing formulations. In addition, it may be promising to study the physiological aspects of the synergistic effect of gut microbiota and *Bt* on the Colorado potato beetle.

## Supporting information

S1 Fig Rarefaction curves of the OTU number for each sample. (PDF)

S2 Fig Sensitivity of symbiotic bacteria and *B. thuringiensis* to an antibiotic. 1 –Disc soaked in antibiotic (amikacin), diameter—10 mm; 2 –zone of inhibition; 3 –bacterial growth.
(PDF)

S3 Fig CFU counts of Enterobacteriaceae in the midgut of CPB larvae at 24 h posttreatment with distilled water (left) and antibiotic (amikacin) 30 mg/L (right).
(PDF)

S1 Appendix (XLSX)
