# Supplementary figures and images for: Influence of Bacillus thuringiensis and avermectins on gut physiology and microbiota in Colorado potato beetle: Impact of enterobacteria on susceptibility to insecticides
Source: PLoS One. 2021 Mar 24;16(3):e0248704. doi: 10.1371/journal.pone.0248704 (PMC7990289; doi:10.1371/journal.pone.0248704)

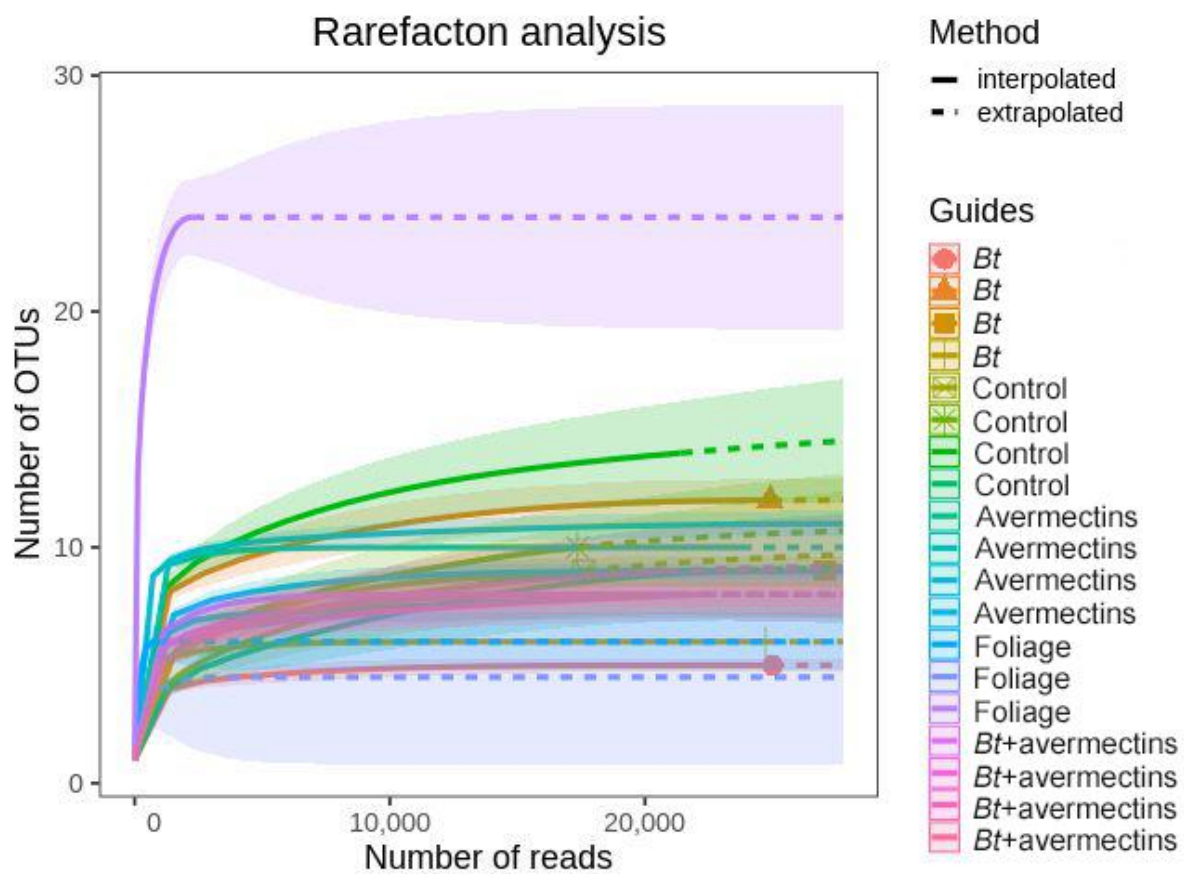

1

2

**S1 Fig. Rarefaction curves of the OTU number for each sample.**

Supplement: S1 Fig — (PDF) [file pone.0248704.s001.pdf]
