# Supplementary material for: Influence of Bacillus thuringiensis and avermectins on gut physiology and microbiota in Colorado potato beetle: Impact of enterobacteria on susceptibility to insecticides
Source: PLoS One. 2021 Mar 24;16(3):e0248704. doi: 10.1371/journal.pone.0248704 (PMC7990289; doi:10.1371/journal.pone.0248704)
Supplement: S2 Fig — 1 –Disc soaked in antibiotic (amikacin), diameter—10 mm; 2 –zone of inhibition; 3 –bacterial growth. (PDF) [file pone.0248704.s002.pdf]

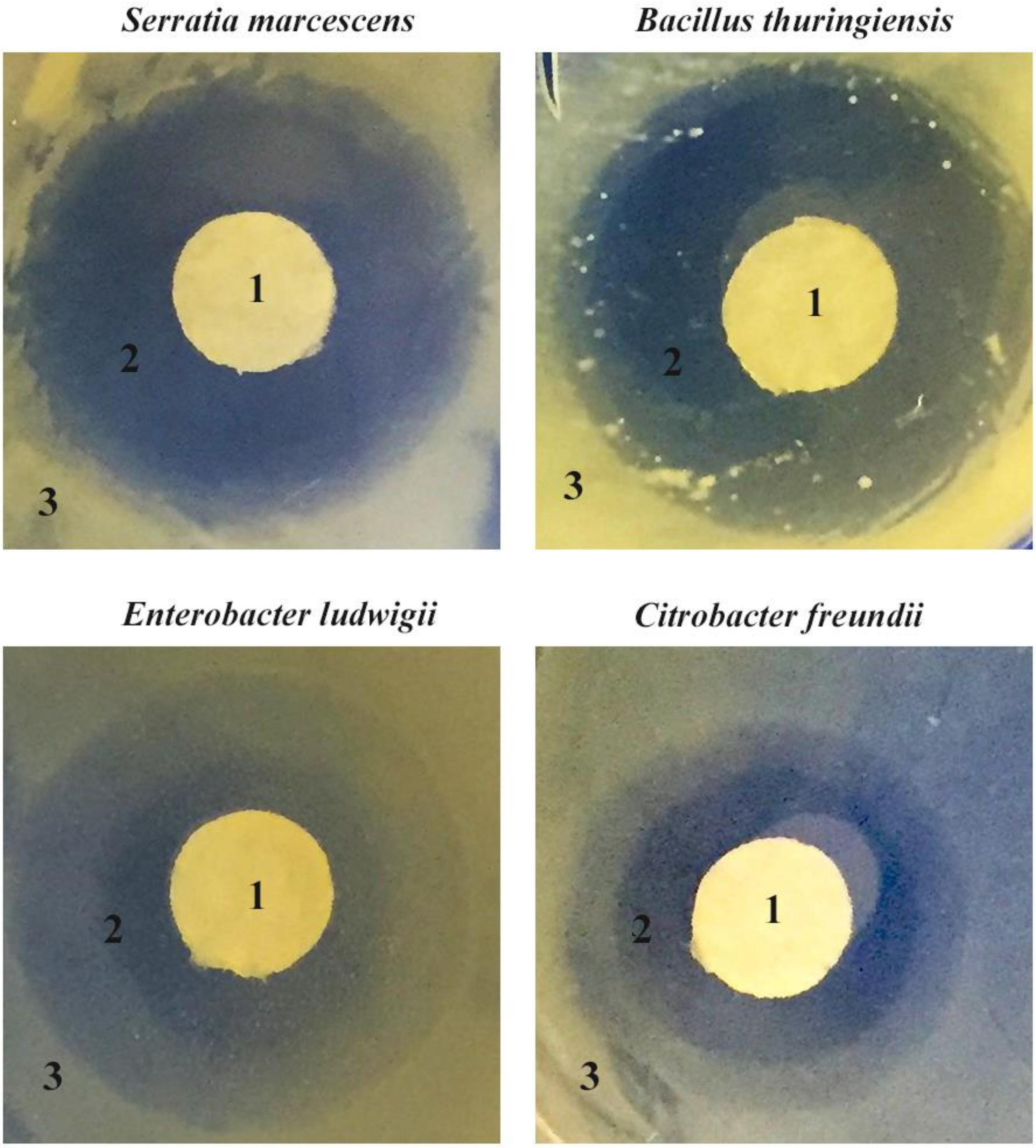

S2 Fig. Sensitivity of symbiotic bacteria and *B. thuringiensis* to an antibiotic. 1 – Disc soaked in antibiotic (amikacin) (diameter – 10 mm); 2 – zone of inhibition; 3 – bacterial growth.
