# Supplementary material for: Influence of Bacillus thuringiensis and avermectins on gut physiology and microbiota in Colorado potato beetle: Impact of enterobacteria on susceptibility to insecticides
Source: PLoS One. 2021 Mar 24;16(3):e0248704. doi: 10.1371/journal.pone.0248704 (PMC7990289; doi:10.1371/journal.pone.0248704)
Supplement: S3 Fig — CFU counts of Enterobacteriaceae in the midgut of CPB larvae at 24 h posttreatment with distilled water (left) and antibiotic (amikacin) 30 mg/L (right). (PDF) [file pone.0248704.s003.pdf]

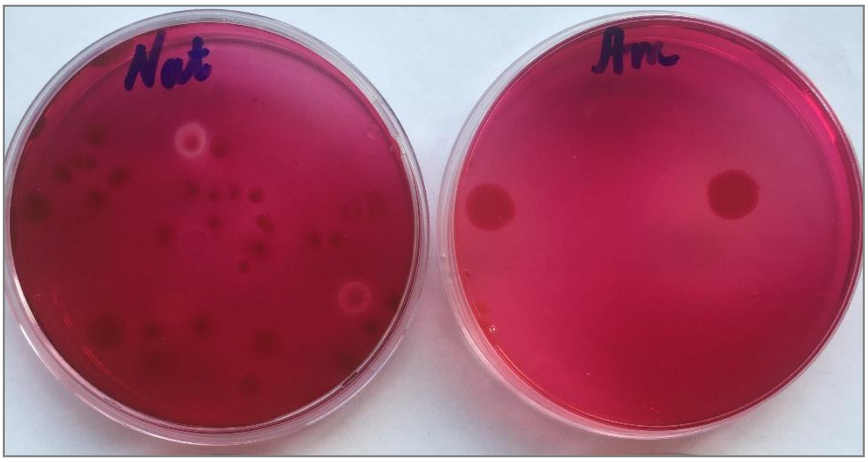

S3 Fig. CFU counts of Enterobacteriaceae in the midgut of CPB larvae at 24 h posttreatment with distilled water (left) and antibiotic (amikacin) 30 mg/L (right).
